# Cardiovascular magnetic resonance imaging in early anthracycline cardiotoxicity

**DOI:** 10.1186/1532-429X-11-S1-P18

**Published:** 2009-01-28

**Authors:** Gillian Smith, Paul Kotwinski, John Paul Carpenter, Montgomery Hugh, Dudley Pennell

**Affiliations:** 1 Royal Brompton Hospital, London, UK; 2 University College London, London, UK

**Keywords:** Breast Cancer, Ejection Fraction, Left Ventricular Ejection Fraction, Cardiovascular Magnetic Resonance, Myocardial Edema

## Introduction

Anthracyclines are the mainstay of adjuvant chemotherapy for patients with breast cancer. This therapy is known to be cardiotoxic with population risk rising with dosage but individual susceptibility is idiosyncratic thus optimal dosage is denied to many. Cardiovascular magnetic resonance (CMR) offers an accurate means by which to assess ventricular function prior to and following chemotherapy. It also offers excellent intrinsic contrast which allows the detection of change at a tissue level which may indicate inflammatory changes without the need for a contrast agent and therefore cannulation. This would be of particular value for monitoring patients undergoing courses of chemotherapy who frequently develop problems with venous access or become needle phobic.

## Purpose

As part of a prospective gene environment study in breast cancer, this study attempts to identify early markers of anthracycline mediated toxicity which may predict later cardiac dysfunction.

## Methods

276 female patients with early breast cancer were scanned before commencement of chemotherapy and one year post completion. CMR was performed using a 1.5 T Siemens Avanto scanner with a 6 channel phased array cardiac coil. Patients were scanned 3 days post first cycle. Cine images are acquired using a retrospectively gated high temporal resolution SSFP sequence. Left ventricular ejection fraction (LVEF) and mass (LVM) are measure using a semi-automated analysis package (LVtools, Cardiovascular Imaging Solutions, London, UK). T2 weighted STIR images were also acquired and signal intensity compared.

## Results

To date, 28 patients have had day 3 scans and one year follow-up studies. Mean ejection fraction (EF) changed from 72.9 ± 5.0% to 71.0 ± 4.6% (p = 0.06). 14 patients showed a significant reduction in ejection fraction (mean 75.5 ± 4.6% to 70.4 ± 4.9%). In these patients, the LVM increased from 115.7 ± 14.6 g to 125.1 ± 16.7 g (p = 0.0002), and this was associated with an increase in STIR image intensity (62.7 ± 19.2 to 71.7 ± 26.9; p = 0.05). Of the 14 patients who showed no reduction in LVEF (70.0 ± 3.9 at baseline vs 71.8 ± 4.4 at follow-up), there was no significant change in either LVM (111.8 ± 13.0 g vs 116.1 ± 11.4 g, p = 0.2) or Stir (64.7 ± 20.6 vs 63 ± 17.1, p = 0.6) at day 3.

## Conclusion

CMR with Stir and left ventricular mass measurements, which reflect myocardial edema, offers a sensitive predictor at day 3 post chemotherapy of myocardial dysfunction after one year secondary to anthracycline cardiotoxicity.

